# Ovarian torsion in an adolescent: A case report and review of diagnostic challenges

**DOI:** 10.1016/j.ijscr.2025.111007

**Published:** 2025-02-03

**Authors:** Aicha Chebil, Yosra Jmaa, Sirine Haouala, Mohamed Ali Chaouch, Fethi Jebali, Hayet Laajili

**Affiliations:** a Department of Gynecology, Hadj Ali Soua Hospital, Ksar Hellal, Tunisia; b Department of Visceral and Digestive Surgery, Monastir University Hospital, Monastir, Tunisia; c Department of Intensive Care and Reanimation B, Monastir University Hospital, Monastir, Tunisia

**Keywords:** Torsion, Fallopian tube, Adolescent, Case report

## Abstract

**Introduction and importance:**

Ovarian torsion is an uncommon but serious cause of acute abdominal pain in children. Due to its nonspecific symptoms and diagnostic difficulties, timely identification and intervention are crucial to preserving ovarian function and future fertility.

**Case presentation:**

A 13-year-old woman presented a 3-day history of pain and vomiting from the right iliac fossa. Clinical examination revealed localized tenderness without abdominal contracture or palpable masses. Laboratory results were unremarkable, and imaging showed a simple 5 cm unilocular ovarian cyst with hyperechoic content and an enlarged right ovary. Laparotomy confirmed a right ovarian torsion with a necrotic ovary. Despite detorsion, no reperfusion was observed, necessitating a right adnexectomy. The postoperative course was straightforward.

**Clinical discussion:**

Ovarian torsion is rare but poses a significant diagnostic challenge due to its variable clinical and radiological presentation. The primary diagnostic tool is ultrasound, often supplemented by Doppler imaging, although the absence of a Doppler signal does not rule out torsion. Differential diagnoses include appendicitis, hemorrhagic cysts, and other conditions that mimic acute abdomen.

**Conclusions:**

Early recognition and management are essential, particularly in young patients, to mitigate long-term reproductive consequences.

## Highlights

• Ovarian torsion is a serious cause of acute abdominal pain in children. It presents significant diagnostic challenges due to nonspecific symptoms, making timely identification and intervention crucial to preserving ovarian function and future fertility.
• The article discusses the case of a 13-year-old girl with a three-day history of right iliac fossa pain and vomiting. Imaging revealed a 5 cm unilocular ovarian cyst and an enlarged right ovary. Laparotomy confirmed ovarian torsion with a necrotic ovary, leading to a right adnexectomy. Despite the detorsion attempt, no reperfusion was observed.
• The diagnosis of ovarian torsion is complex due to its variable clinical and radiological presentations. Ultrasound, supplemented by Doppler imaging, is the primary diagnostic tool, though the absence of a Doppler signal does not rule out torsion. Early recognition and surgical intervention are essential to prevent long-term reproductive complications.

## 1 Introduction and importance

Ovarian torsion is an uncommon but serious cause of acute abdominal pain in children, occurring less frequently than rupture of the ovarian cyst or intracystic hemorrhage [1]. It results from spontaneous twisting of the vascular and lymphatic pedicle of the adnexa, leading to a spectrum of pathological changes ranging from ovarian congestion to hemorrhagic infarction. The nonspecific clinical presentation and the overlap of symptoms with other acute abdominal conditions make the diagnosis particularly challenging, often leading to delays in intervention [[2], [3]]. Early and accurate diagnosis is crucial, as prolonged torsion can lead to irreversible ovarian necrosis, significantly affecting future fertility. However, preoperative identification remains difficult, as imaging findings are often inconclusive, and ultrasound and Doppler studies sometimes fail to confirm torsion definitively. As a result, the diagnosis is often established only at the time of surgical exploration [[2], [3]]. Unfortunately, in pediatric patients, conservative treatment is rarely feasible due to delay in diagnosis and the extent of ovarian damage at the time of surgical intervention. This case report, presented according to the SCARE guidelines [3], underscores the clinical and diagnostic challenges associated with adolescent ovarian torsion. By highlighting a typical presentation, it aims to reinforce the need for greater awareness and early surgical exploration in suspected cases to optimize ovarian preservation and improve reproductive outcomes.

## 2 Case presentation

**Fig. 1:**
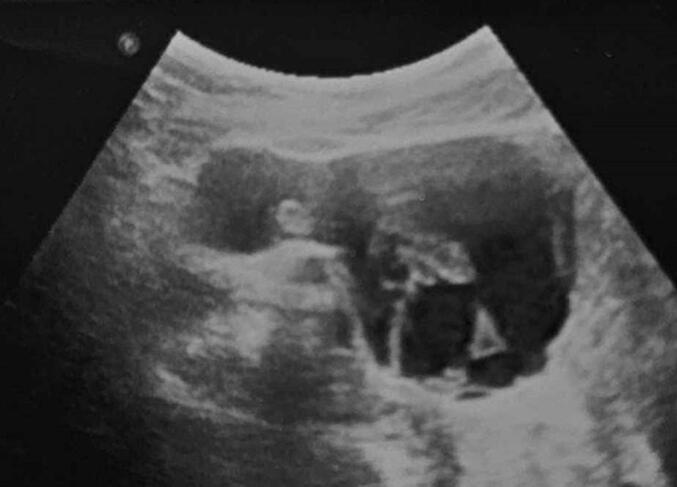
Ultrasonography view showing a simple 5 cm unilocular ovarian cyst with hyperechoic content with an enlarged right ovary.

**Fig. 2:**
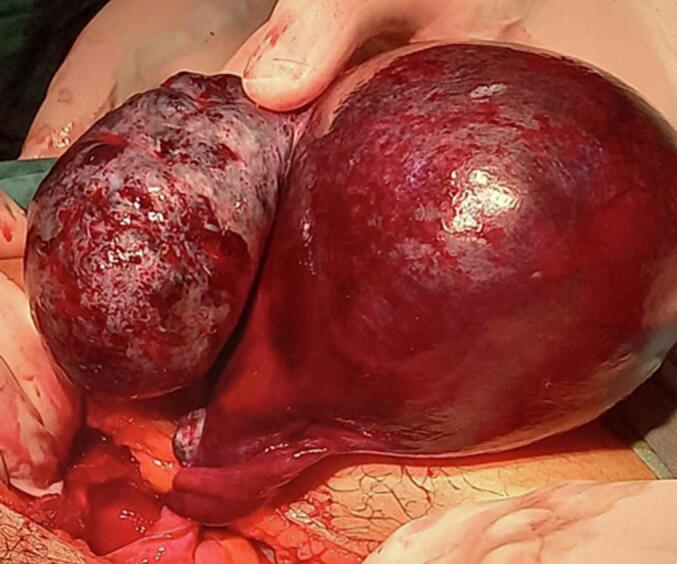
Intraoperative view of the right ovarian cyst with two turns and a necrotic ovary.

A 13-year-old girl, without significant medical or surgical history, presented with a three-day history of acute right iliac fossa pain associated with intermittent vomiting. She was afebrile, had regular menstrual cycles since menarche at the age of 12, and reported being on the 15th day of her cycle. She had no urinary or gastrointestinal symptoms. At admission to the emergency department, her vital signs were stable, with a body temperature of 36 °C and a blood pressure of 110/70 mmHg. Physical examination revealed localized tenderness in the right iliac fossa without signs of peritoneal irritation, guarding, or palpable mass. Rectal examination was unremarkable and gynecological assessment confirmed an intact hymen without vaginal bleeding. Laboratory tests showed no signs of infection or inflammation (C-reactive protein = 5 mg/L, leukocyte count = 9300/mm^3^). The urine pregnancy test was negative and the urinalysis was normal. Abdominal radiography did not show significant findings, especially no air-fluid levels suggestive of bowel obstruction. A pelvic ultrasound with a full bladder revealed a normal uterus and a left ovary. The right ovary appeared to be enlarged, with a simple unilocular cyst of 5 cm in diameter and hyperechoic content (Fig. 1). Given the clinical and radiological findings, a diagnosis of right ovarian torsion was suspected and surgical intervention was decided. Laparotomy was performed instead of laparoscopy due to suspicion of ovarian necrosis and concerns about potential intraoperative complications. Intraoperative findings confirmed the right ovarian cyst measuring 5 cm in its largest dimension, with two complete twists of the pedicle, leading to ovarian ischemia (Fig. 2). The ovary appeared markedly enlarged and necrotic. After detorsion, no signs of reperfusion were observed despite a 10-min waiting period. Given the lack of viability, a right adnexectomy was performed. The Appendix was examined and found to be healthy. The left ovary and fallopian tube were normal. The total surgical time was 45 min, with an estimated blood loss of 50 ml. The postoperative course was uneventful and the patient was discharged on postoperative day 2 without complications. At the one-month follow-up, she remained asymptomatic, with no signs of postoperative infection or complications. She was advised on the implications of unilateral oophorectomy for future fertility and the importance of long-term gynecological follow-up.

## 3 Case discussion

Ovarian torsion is a rare event that can occur at any age, from the fetal period to adulthood [1]. It is a rare but potentially serious cause of acute abdominal pain in children [2,3]. This condition results from spontaneous rotation of the vascular and lymphatic pedicle of the ovarian adnexa around its axis, which presents morphological variability that varies from ovarian edema to hemorrhagic infarction [2]. Due to the nonspecificity of clinical symptoms and assessment challenges in children, the diagnosis of ovarian torsion remains difficult to establish [2]. Radiological aspects are also variable, highlighting the importance of a radioclinical correlation for the radiologist in the diagnostic process [4]. In most cases, surgical exploration remains essential to confirm the diagnosis and manage this surgical emergency. As in adults, ovarian torsion in children usually occurs in the presence of a pathological ovary, often associated with an adnexal mass or cyst. However, it can also occur in a structurally normal ovary [6]. The usual predisposing factor is an ipsilateral adnexal mass that is almost always benign. Studies indicate that an ipsilateral adnexal mass, most commonly benign, is a primary predisposing factor. The risk of torsion is particularly correlated with cyst size, increasing significantly when cyst diameter exceeds 4 to 5 cm [5]. Paratubal torsion in children includes acute abdominal pain, which can mimic other common pediatric conditions such as appendicitis (8). Clinical evaluation is of paramount importance in the diagnosis of isolated tubal torsion. Identifying a similar self-limiting painful episode is crucial. These episodes of subtortion must be sought during interrogation, dating back to previous weeks or months (9). In addition, the sudden onset of symptoms is also significant. Pain is often in the lower abdomen or flank and may be accompanied by abdominal tenderness with or without peritoneal signs and is frequently accompanied by one or more episodes of nausea and vomiting. Subsequently, pain usually progresses in attacks, either subacutely with a reduction in symptoms, which can make diagnosis and treatment more difficult to establish, or acutely, where the surgical indication becomes more obvious. Furthermore, physical examinations may reveal adnexal tenderness during rectal examinations, but a mass may not be palpable. Biological examinations are of little contribution. However, a discreet leukocytosis can be observed in contrast with the absence of fever. The radiological appearance of ovarian torsion is very variable and is related to the degree of internal hemorrhage, stromal edema, infarction, and necrosis that occur at the time of diagnosis. However, the most common radiological appearance of ovarian torsion in young girls is unilateral enlargement of the ovary [8]. An enlarged ovary can appear as a solid hyperechoic structure often associated with peripheral cysts that produce the pearl necklace appearance [9]. This appearance constitutes a major diagnostic criterion, but often the appearance is nonspecific. An adnexal mass increases the volume of the ovary and therefore theoretically predisposes it to torsion [8]. Cystic, follicular, and paratubal lesions, particularly simple, were the adnexal lesions most frequently encountered in ovarian torsions [6]. Generally, it is a homogeneous mass at the beginning and then heterogeneous at a more advanced stage, reflecting the presence of areas of hemorrhagic necrosis [10]. Walls often appear thickened by epithelial edema. Indeed, the presence of an arterial Doppler signal within the mass does not exclude the diagnosis, and its absence is not a specific sign of ovarian torsion, but increases the specificity of the diagnosis, combined with the clinic and ultrasound [2]. Furthermore, CT and magnetic resonance imaging do not appear to allow earlier and more discriminating diagnoses [11]. Among differential diagnoses, appendicitis is the main one to consider in the right place, but several other conditions can mimic ovarian torsions, such as hemorrhagic cysts, urinary infection, renal colic, tuboovarian abscesses, ectopic pregnancy, and tumor lesions [8]. The diagnosis of painful ovulation will be considered last. However, a combined evaluation of the clinical context, biological data, and radiological results generally allows for an accurate diagnosis. Therapeutically, the main objective of urgent treatment of ovarian torsion is to preserve ovarian function, particularly fertility. This involves emergency laparoscopic or laparotomic surgical derotation with visual evaluation of ovarian viability. Salpingo-oophorectomy is generally considered in cases of severely necrotic ovaries.

## 4 Conclusions

Ovarian torsion represents a diagnostic and therapeutic emergency because any delay in its treatment can lead to ischemia, then ovarian necrosis, thus increasing the risk of fertility complications. This rare and serious in children is the result of the spontaneous rotation of the vascular and lymphatic pedicle of the ovarian adnexa around its axis, which can cause a variety of manifestations ranging from ovarian edema to hemorrhagic infarction, jeopardizing the viability of the ovary and future fertility. Although radiological presentations may vary, ultrasound coupled with Doppler makes it possible to establish the diagnosis in most cases in the presence of a suggestive clinical picture. Surgery is the method of choice to confirm the diagnosis and allow conservative intervention when possible.

## Patient consent

Written informed consent was obtained from the patient to publish this case report and accompanying images. On request, a copy of the written consent form is available for review by the editor-in-chief of this journal.

## Ethical approval

For all case reports, ethical approval is exempt/waived at Monastir University Hospital.

## Funding

No funding.

## Guarantor

Mohamed Ali Chaouch.

## Research registration number

Not applicable.

## Provenance and peer review

Not commissioned, externally peer reviewed.

## Conflict of interest statement

The authors declare no conflict of interest.
